# Jaundice and Elevated Beta-Human Chorionic Gonadotropin in a Female: Duodenal Choriocarcinoma, Not Pregnancy

**DOI:** 10.7759/cureus.8336

**Published:** 2020-05-28

**Authors:** Nicholas A Berry, Bruce Berry, Andrew C Berry

**Affiliations:** 1 Internal Medicine, Mayo Clinic School of Medicine, Scottsdale, USA; 2 Internal Medicine, Froedtert & the Medical College of Wisconsin, Milwaukee, USA; 3 Gastroenterology, Larkin Community Hospital, South Miami, USA

**Keywords:** choriocarcinoma, adenocarcinoma, duodenal cancer, biliary, elevated liver associated enzymes

## Abstract

A 29-year-old woman with developmental delay presented with 2.5 weeks of jaundice of the skin with accompanying microcytic anemia (hemoglobin 6.8 g/dL, mean corpuscular volume 70.5 fL), elevated liver enzymes (aspartate aminotransferase 77 U/L, alanine aminotransferase 95 U/L, alkaline phosphatase 362 U/L), total bilirubin (9.5 mg/dL; 4.4 mg/dL direct), lipase (325 U/L), and cancer antigen 19-9 (68 U/mL). The patient had no prior gastrointestinal or liver disease. CT of the chest/abdomen/pelvis found a large lobulated non-fully obstructing mass in the second and third part of the duodenum, with endoscopic biopsies yielding an invasive, well-differentiated adenocarcinoma positive for cytoplasmic-stained cells to antibody to beta-human chorionic gonadotropin (hCG) antigen, suggesting a duodenal choriocarcinoma. Treatment included biliary drainage with a percutaneous transhepatic catheter and folinic acid, fluorouracil, and oxaliplatin (FOLFOX) chemotherapy, but a repeat CT scan five months later revealed an increase in tumor size and invasion; the patient died shortly thereafter. Beta-hCG-secreting choriocarcinomas are rare, rapidly growing, highly invasive malignant tumors and are uncommonly present at extragonadal sites.

## Introduction

Beta-human chorionic gonadotropin (hCG)-secreting choriocarcinomas are rare, rapidly growing, highly invasive malignant tumors that present most often in the uterus after pregnancy or abortion, as they are remnants from trophoblastic or totipotent germ cells [1,2]. Extragonadal sites that have been reported include the gastrointestinal tract, liver, lung, breast, prostate, urinary bladder, and nose [2,3]. Primary extragonadal choriocarcinomas are very rare, with the majority within or along with adenocarcinomas of the organ in question [2,3]. Presenting symptoms may range from end-stage malignancy with obstructive and metastatic symptoms to common complaints such as pruritus, jaundice, and overt or occult anemia. Although multiple theories regarding the histogenesis of duodenal choriocarcinomas exist, the highly invasive nature of this tumor warrants timely evaluation, as many patients are diagnosed in the metastatic stage [2].

## Case presentation

A 29-year-old woman with developmental delay presented with 2.5 weeks of jaundice of the skin. The patient was found to have severe microcytic anemia (hemoglobin 6.8 g/dL, mean corpuscular volume 70.5 fL), elevated liver enzymes (aspartate aminotransferase 77 U/L, alanine aminotransferase 95 U/L, alkaline phosphatase 362 U/L), total bilirubin (9.5 mg/dL; 4.4 mg/dL direct), lipase (325 U/L), and cancer antigen 19-9 (68 U/mL). Pregnancy tests revealed elevated serum and urine beta-hCG at a serum level of 140 mIU/mL (in a non-pregnant female <5.0 mIU/mL). Subsequent ultrasound showed no intrauterine pregnancy.

**Figure 1:**
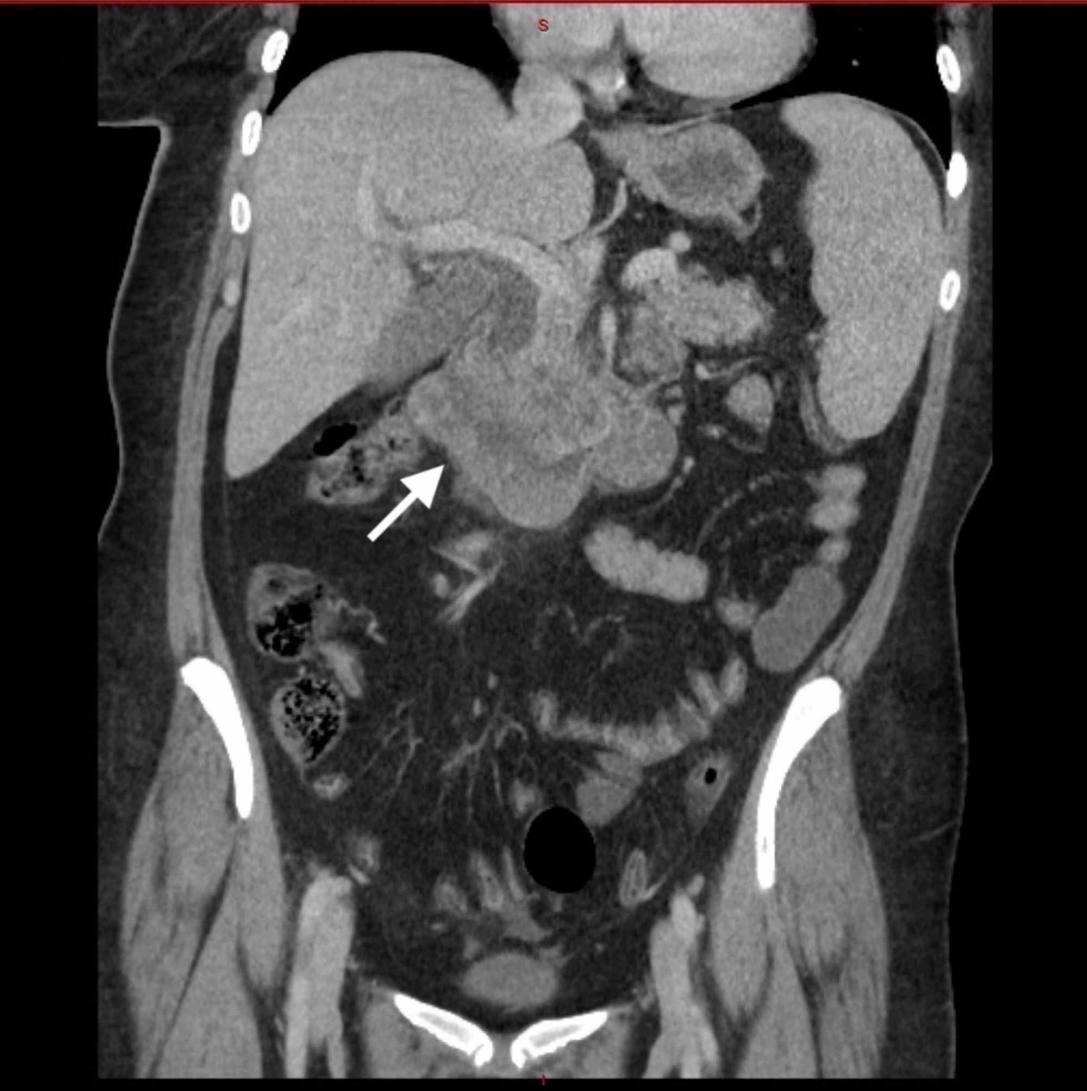
CT of the chest/abdomen/pelvis with intravenous contrast revealed a large, non-obstructing, lobulated mass in the second and third part of the duodenum The large 7.5 x 5.0 x 7.0 cm lobulated, non-obstructing, duodenal mass is seen displacing the head of the pancreas superiorly and anteriorly (arrow). An adjacent satellite lesion to the left of the dominant mass measures 3.5 x 2.5 cm, with two regional lymph nodes in the adjacent bowel mesentery. The dominant mass abuts the inferior vena cava and aorta. Both pancreatic duct dilation (6 mm at site of pancreatic head) and common bile duct dilation (15 mm), with accompanying intrahepatic duct dilation, are shown.

**Figure 2:**
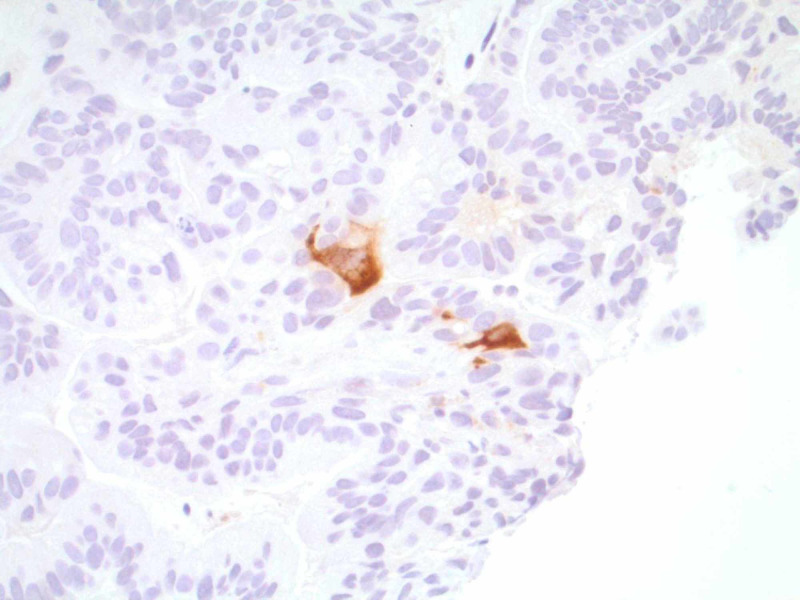
Cytoplasmic-stained cells with antibody to beta-hCG antigen, suggesting a choriocarcinoma hCG, human chorionic gonadotropin. Under 40X magnification; tumor sequencing found 14 genomic mutations, including KRAS and TP53.

**Figure 3:**
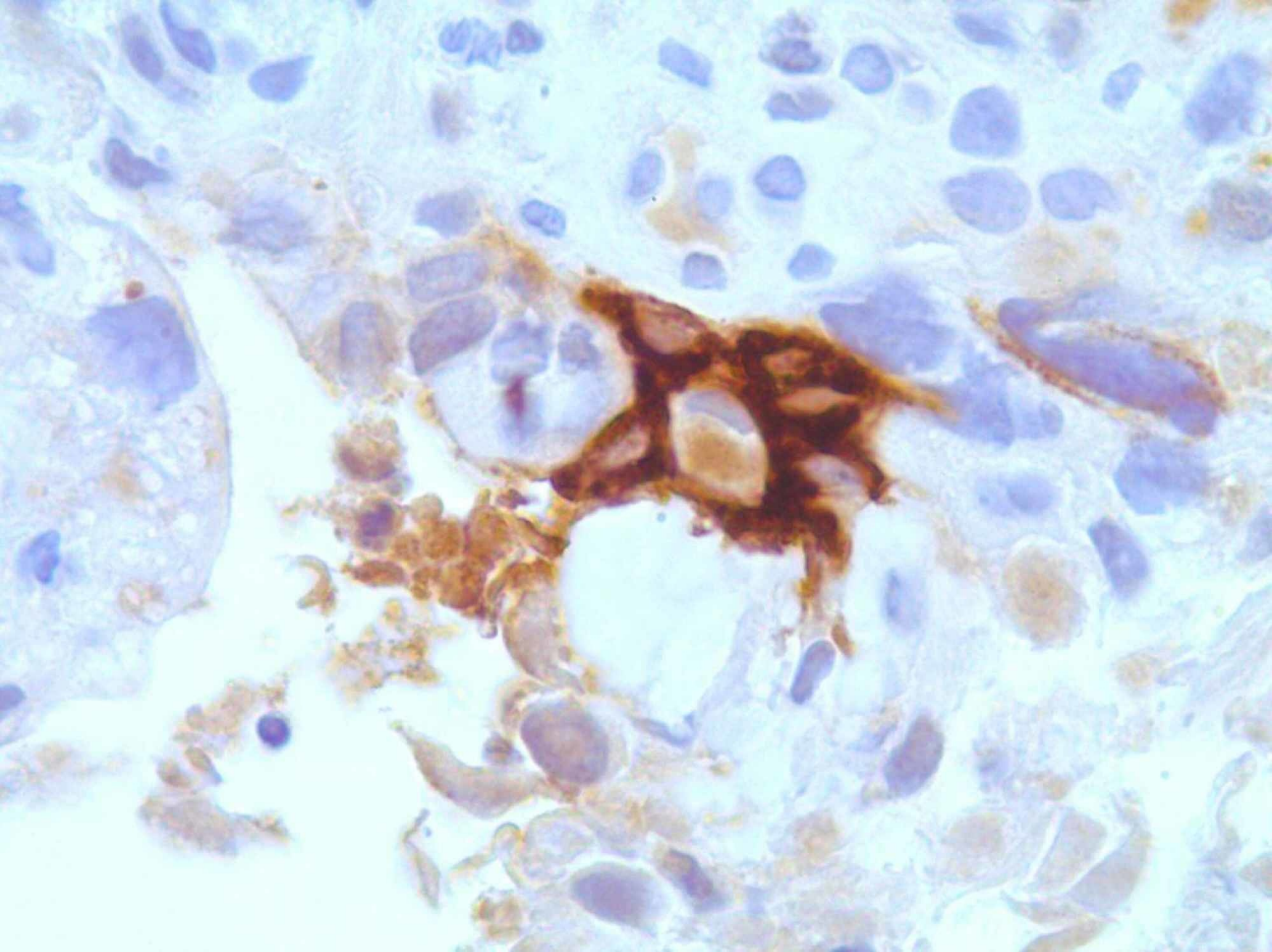
Cytoplasmic-stained cells with antibody to beta-hCG antigen, suggesting a choriocarcinoma hCG, human chorionic gonadotropin. Under 100X magnification; tumor sequencing found 14 genomic mutations, including KRAS and TP53.

CT of the chest/abdomen/pelvis with intravenous contrast revealed a large (7.5 x 5.0 x 7.0 cm), non-obstructing, lobulated mass in the second and third part of the duodenum, displacing the head of the pancreas superiorly and anteriorly (Figure 1). An adjacent satellite lesion to the left of the dominant mass measured 3.5 x 2.5 cm, with two regional lymph nodes in the adjacent bowel mesentery. Both pancreatic duct dilation (6 mm at the site of pancreatic head) and common bile duct dilation (15 mm), with accompanying intrahepatic duct dilation, are shown (Figure 1). Upper endoscopy with biopsies was later performed. Biopsy diagnosis revealed an invasive, well-differentiated adenocarcinoma with cytoplasmic-stained cells with antibody to beta-hCG antigen, suggesting a choriocarcinoma (Figures 2, 3). Treatment included biliary drainage with a percutaneous transhepatic catheter and folinic acid, fluorouracil, and oxaliplatin (FOLFOX) chemotherapy. A CT scan done five months later revealed an increase in duodenal tumor size with invasion into the pancreas and right hepatic lobe. Palliative radiotherapy was initiated, but the patient died several months later.

## Discussion

We present the case of a never-pregnant female with sole complaints of jaundice who was found to have a large, lobular duodenal adenocarcinoma with immunohistochemical analysis positive for a rare beta-hCG-secreting duodenal choriocarcinoma. Multiple theories regarding the histogenesis of duodenal choriocarcinomas exist. One choriocarcinoma theory suggests that remnant totipotent stem cells differentiate into neoplastic beta-hCG-secreting cells [4]. Another choriocarcinoma theory, called the dedifferentiation theory, states that malignant adenocarcinoma cells dedifferentiate into ectodermal cells and then re-differentiate into beta-hCG-secreting trophoblastic cells [4]. In the presented case, it is possible that one or more duodenal adenocarcinoma cells carrying the tumor’s known genomic alterations dedifferentiated and then re-differentiated to produce beta-hCG-secreting cells. It remains imperative to obtain thorough immunohistochemical analysis to adequately make a diagnosis, as many times it is obtained solely via surgical specimens only, and not biopsy specimens [2]. Serologic markers serve as an adjunct diagnostic and treatment monitoring modality; however, they cannot be the sole evaluation tool. The carcinoembryonic antigen (CEA) level can be modestly elevated in the setting of concomitant adenocarcinoma, as the choriocarcinomatous component is CEA negative [2].

Regarding the gastrointestinal tract, patients may present with anemia or bleeding from the ulcerated mass, mimicking peptic ulcer disease [3]. Our patient’s advanced duodenal tumor caused biliary obstruction-induced jaundice as initial presentation, with subsequent radiographic pancreatic and biliary duct obstruction. Management begins with identifying the source of jaundice and anemia. If the tumor cannot be medically treated with chemotherapy, more palliative radiotherapy-induced tumor reduction can relieve pain, mitigate blood loss, and reverse gastrointestinal luminal, biliary, and pancreatic obstruction. There is no overt consensus regarding therapeutic strategies, as the histogenetic pathway is still debatable. Oftentimes, chemotherapies used for gestational choriocarcinoma are utilized [5]. Those with solely choriocarcinoma components have been treated with methotrexate regimens [6]. Moreover, treatment regimens, such as ours with FOLFOX, have also targeted the adenocarcinoma component of the small bowel tumor [6-8]. No human epidermal growth factor receptor 2 (HER-2) positivity was seen in our case, but some reports of HER-2-positive cases to date have shown a positive adenocarcinoma or choriocarcinoma component response with trastuzumab combined with docetaxel and carboplatin [4]. There remains a paucity of documented cases with gastrointestinal choriocarcinoma tumors to clearly guide treatment algorithms. Thus, it remains imperative to monitor the case-by-case response to scientifically guided therapies. Our patient initially was given chemotherapeutics; however, both clinical and radiographic data on subsequent monitoring eventually led to more palliative care prior to the patient’s fatal decline.

## Conclusions

While it remains imperative to consider common diagnosis in the differential diagnosis, it remains prudent to also consider atypical disease entities when numerous clinical evaluations and testing modalities are equivocal. Jaundice, elevated beta-hCG, and female gender - in a young individual - all suggest pregnancy as the leading diagnosis. However, with a negative pregnancy workup, clinical anemia, and later suspicious endoscopic findings, one must consider more atypical diagnoses. Our case reinforces the importance of a multi-disciplinary approach to a difficult patient diagnostic workup, coordinating the specialty-specific care of radiologists, endoscopists, and pathologists with the primary medical care team.
